# Characteristic effect of hydroxyurea on the higher-order structure of DNA and gene expression

**DOI:** 10.1038/s41598-024-64538-y

**Published:** 2024-06-15

**Authors:** Haruto Ogawa, Takashi Nishio, Yuko Yoshikawa, Koichiro Sadakane, Takahiro Kenmotsu, Tomoyuki Koga, Kenichi Yoshikawa

**Affiliations:** 1 Faculty of Life and Medical Sciences, Doshisha University, Kyoto, 610-0394 Japan; 2 Cluster of Excellence Physics of Life, TUD Dresden University of Technology, 01307 Dresden, Germany; 3 Department of Molecular Chemistry and Biochemistry, Faculty of Science and Engineering, Doshisha University, Kyoto, 610-0321 Japan; 4 Center for Integrative Medicine and Physics, Institute for Advanced Study, Kyoto University, Kyoto, 606-8501 Japan

**Keywords:** Applied physics, Biological physics, Chemical physics, Statistical physics, thermodynamics and nonlinear dynamics, Techniques and instrumentation, Biochemistry, Chemical biology, Medicinal chemistry, Physical chemistry, Polymer chemistry, Molecular biophysics, Single-molecule biophysics, Biophysical chemistry, DNA, Biophysical methods, Gene expression analysis, Biophysical chemistry, DNA, Pharmacology, Target validation, Medical genetics, Chemical biology, Molecular medicine

## Abstract

Hydroxyurea (HU; hydroxycarbamide) is a chemotherapy medication used to treat various types of cancer and other diseases such as sickle cell anemia. HU inhibits DNA synthesis by targeting ribonucleotide reductase (RNR). Recent studies have suggested that HU also causes oxidative stress in living systems. In the present study, we investigated if HU could directly affect the activity and/or conformation of DNA. We measured in vitro gene expression in the presence of HU by adapting a cell-free luciferase assay. HU exhibited a bimodal effect on gene expression, where promotion or inhibition were observed at lower or higher concentrations (mM range), respectively. Using atomic force microscopy (AFM), the higher-order structure of DNA was revealed to be partially-thick with kinked-branching structures after HU was added. An elongated coil conformation was observed by AFM in the absence of HU. Single DNA molecules in bulk aqueous solution under fluctuating Brownian motion were imaged by fluorescence microscopy (FM). Both spring and damping constants, mechanical properties of DNA, increased when HU was added. These experimental investigations indicate that HU directly interacts with DNA and provide new insights into how HU acts as a chemotherapeutic agent and targets other diseases.

## Introduction

Hydroxyurea (HU), or hydroxycarbamide, is an antitumor drug^1–5^ used to treat chronic myeloid leukemia and certain types of head and neck cancer^6,7^. HU is also the primary drug of choice for the treatment of sickle cell anemia^3,7,8^. A promising new indication for HU in treating Alzheimer's disease has recently attracted much attention for its ability to prevent cognitive decline^9^.

Antitumor activity of HU was first reported in the 1960s^6,10^. HU inactivates ribonucleotide reductase (RNR), causing a decrease in the cellular pool of deoxyribonucleoside triphosphates, which leads to the inhibition of DNA synthesis^11–16^. Recently, experiments using budding and fission yeasts reported that HU globally inhibited RNA synthesis and transcription by RNA polymerase^17^. Therefore, the cytotoxic and antitumor activities of HU have been attributed to enzyme-mediated effects. However, little work has been conducted on the direct interaction of HU with DNA and its effects on gene expression. Even though HU is actively used medically to treat many diseases, the exact mechanism of how HU works is not fully known at present.

To gain further insight into the exact biological mechanism of the action of HU, here we explored if HU could directly affect DNA activity (i.e., gene expression) and/or DNA conformation (i.e., DNA structure). We used an in vitro gene expression luciferase assay to measure DNA activity in the presence of HU. Interestingly, we found that HU exhibited a bimodal effect on gene expression. Promotion was observed at lower concentrations of HU (< 10 mM), while inhibition of gene expression was discovered at higher concentrations (> 10 mM). In addition, a single molecular observation of genome-size DNA by atomic force microscopy (AFM) and fluorescence microscopy (FM) revealed characteristic changes in the higher-order structures and the viscoelasticity of a single DNA molecule depending on the concentration of HU. These results clearly indicate the direct effect of HU on the higher-order structure of DNA. We discuss the bimodal effect of gene expression in relation to the conformational change of DNA.

## Results

### Effect of HU on the efficiency of gene expression

**Figure 1:**
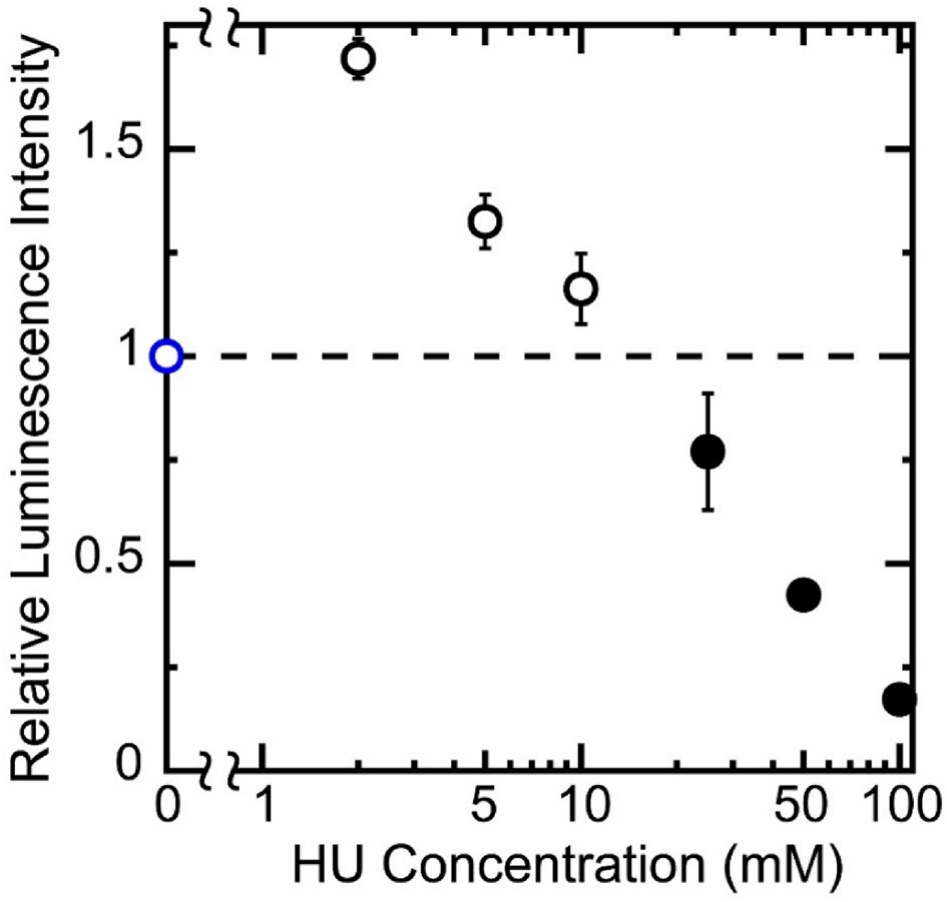
The effect of HU on gene expression efficiency. The vertical axis is the relative emission intensity of the luciferin-luciferase reaction. The horizontal axis is the HU concentration. The DNA (luciferase T7 control DNA) concentration was 0.6 µM in nucleotide unit. Each experiment was repeated independently at least three times. Data are presented as the mean ± SD.

We explored the effect of HU on the activity of gene expression by adapting an in vitro cell-free luciferase assay with TnT (Rabbit Reticulocyte Lysate) T7 Quick Coupled Transcription/Translation System. Figure 1 shows the relative luminescence intensity of the luciferin-luciferase reaction at various concentrations of HU. The intensity was normalized to the control experiment observed in the absence of HU. Interestingly, the results indicate that HU has a bimodal effect where enhancement and inhibition of gene expression were observed at lower and higher concentrations, respectively. The gene activity is ca. 1.7 times higher at 2 mM HU compared to that of control (0 mM HU). At 100 mM HU, the activity is largely depressed, ca. 1/5 to that of control.

### Effect of HU on the higher-order structure of DNA as revealed by atomic force microscopy (AFM)

**Figure 2:**
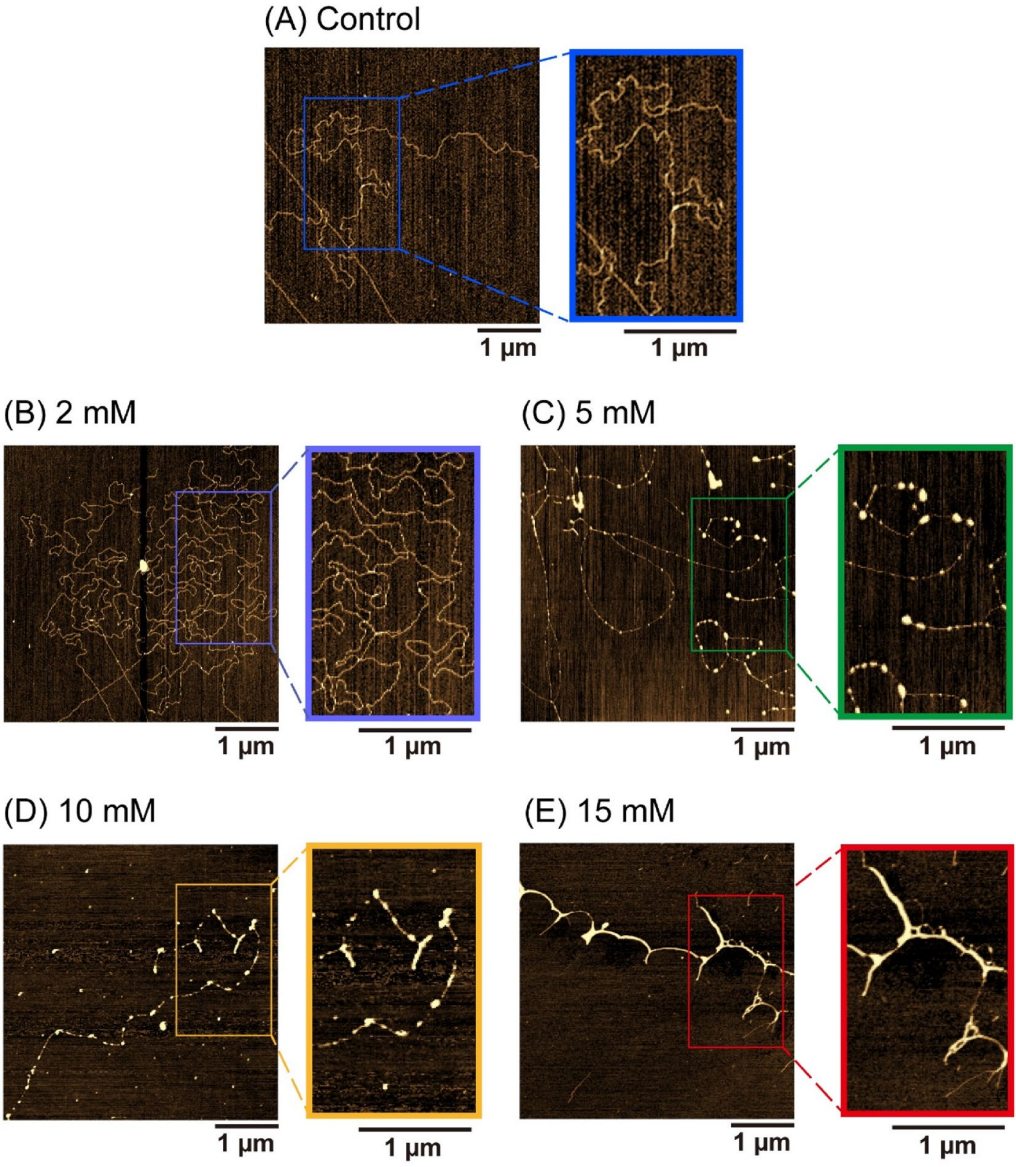
AFM images of T4 GT7 DNA. Representative images of (**A**) Control (0 mM HU), (**B**) 2 mM HU, (**C**) 5 mM HU, (**D**) 10 mM HU, and (**E**) 15 mM HU. Expanded images are shown on the right side for each concentration.

As mentioned above, HU caused a bimodal effect on gene expression. We imaged single DNA molecules using atomic force microscopy (AFM) with a nm-scale resolution to clarify how HU may influence the higher-order structure of T4 GT7 DNA (166 kbp). Figure 2 shows representative AFM images of DNA molecules adsorbed on a mica surface in the presence of different concentrations of HU (2, 5, 10, 15 mM) compared with the control (0 mM HU) (see also Fig. S1). DNA specimens were stably attached to a mica surface using a Tris–HCl buffer (pH 7.4) containing 2 mM MgCl_2_ as previously described^18–20^. DNA shrunk slightly at 2 mM HU (Fig. 2B) in contrast to the elongated conformation in the absence of HU (Fig. 2A). As the concentration of HU increased, partially-thick and kinked-branching structures appeared (Fig. 2C,D) and the kinking conformation pronounced at 15 mM HU (Fig. 2E). These results reveal that HU directly affects the higher-order structure of DNA, which may concern with the bimodal effect (i.e., promotion-inhibition) of gene expression caused by HU.

### Effect of HU on the viscoelasticity of single DNA Molecules observed by fluorescence microscopy (FM)

**Figure 3:**
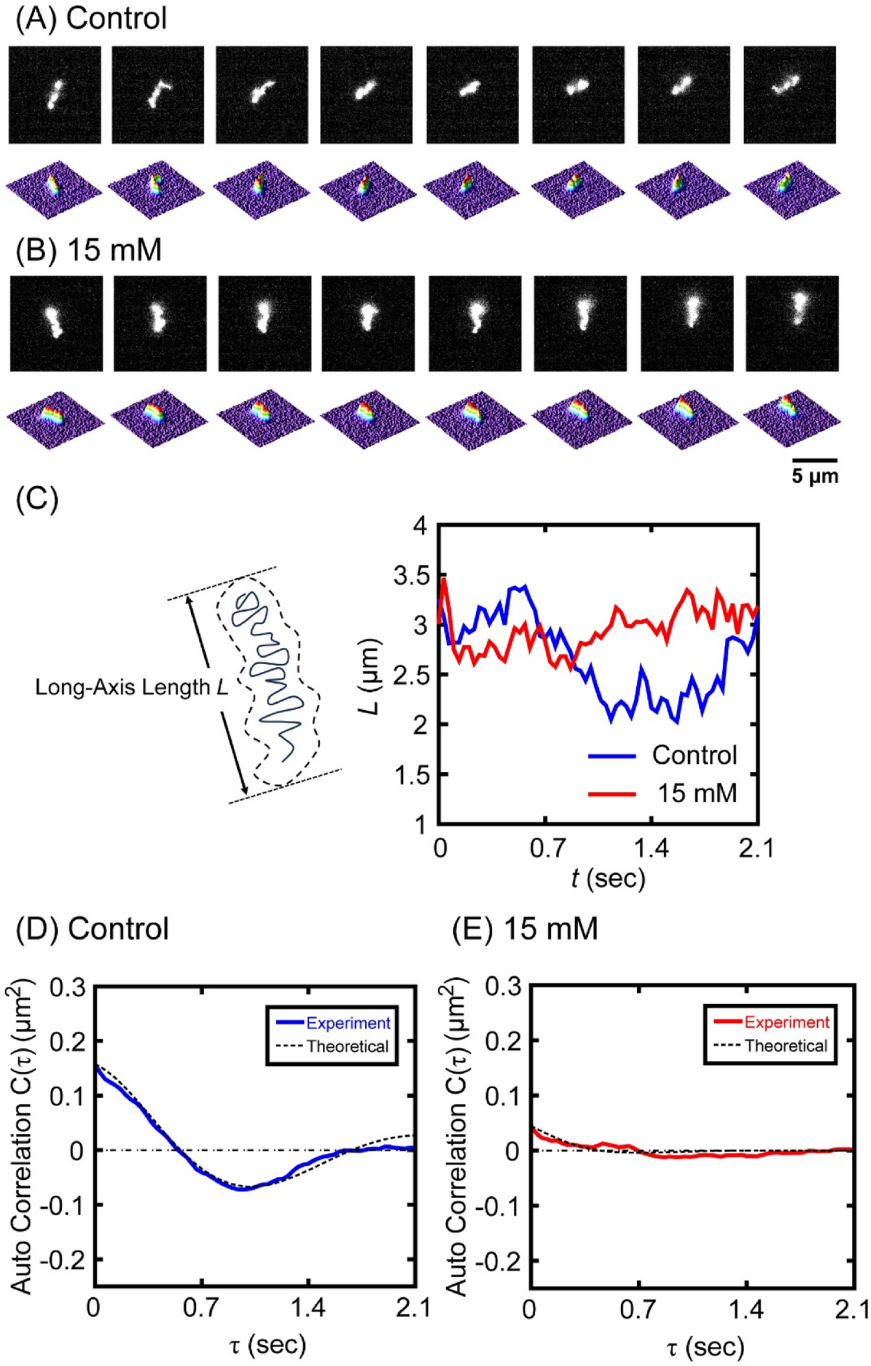
Time-dependent fluctuation of single T4 GT7 DNA molecules under Brownian motion observed by fluorescence microscopy (FM). (**A**) Control (0 mM HU) and (**B**) 15 mM HU treated DNA. The time interval between neighboring frames is 0.3 s. The corresponding quasi-three-dimensional profiles of the fluorescence intensity distribution are shown on the lower frames of each image. Also see Fig. S3. (**C**) Left, schematic representation of the long-axis length *L* for the FM image of a single DNA molecule. Right, time-dependent changes in *L* of T4 GT7 DNA molecules. The time trace lines are DNA without HU (blue, Control) and DNA with 15 mM HU (red). (**D**, **E**) Autocorrelation of the time-dependent fluctuation of the long-axis length of single T4 GT7 DNA molecules. The fitting curves were depicted based on Eq. 2. Fluctuations were measured in the Tris–HCl buffer solution without HU (Control) and with 15 mM HU. At each condition, independent measurements for the fluctuation of single DNA observations by FM were performed at least three times.

Next, we observed real-time Brownian motion of single T4 GT7 DNA molecules in bulk aqueous solution using fluorescence microscopy (FM). FM image examples of individual DNA molecules under intrachain Brownian motion of single DNAs in the aqueous solution without (Fig. 3A) and with 15 mM HU (Fig. 3B). Figure 3C shows time-dependent changes of the long-axis length,* L*, measured from each frame of the single DNA molecule FM movies. The degree of the Brownian fluctuation of DNA was depressed with the addition of 15 mM HU. To quantitatively evaluate the viscoelastic properties of DNA molecules from these data, we performed further numerical steps based on the previously reported analysis methodology^21–23^. First, we evaluated the autocorrelation C(τ) from the time-dependent changes of *L*:

1 $$\begin{array}{*{20}c} {C\left( \tau \right) = \left\langle {L\left( \tau \right) - \bar{L}} \right\rangle \left\langle {L\left( 0 \right) - \bar{L}} \right\rangle } \\ \end{array}$$

where $\overline{L }$ is the time-average of *L*, τ is the time lag between data points, and the symbol, <  > , means the average of the time-dependent variable. Figure 3D,E shows the calculated autocorrelation function (see also Fig. S2). Based on a simple theoretical model of fluctuation–dissipation theory for the thermal fluctuations under harmonic potential, the autocorrelation function is expressed as in Eq. (2)^24,25^:

2 $$\begin{array}{*{20}c} {C\left( \tau \right)\sim \frac{{k_{B} T}}{k}e^{{ - \gamma \tau }} \cos \omega \tau } \\ \end{array}$$

where ${k}_{B}$ is the Boltzmann constant, *T* is the absolute temperature (297 K in our observations), *k* (N/m) is the spring constant, γ (sec^-1^) is the damping constant, and ω is the angular frequency. Considering the relationship *k* ≈ $\frac{{k}_{B}T}{C(0)}$, where C(0) is the value at τ = 0, the spring constant *k* can be evaluated from the initial value of the autocorrelation function. The given fitting curve based on Eq. (2) is shown with a broken line (Fig. 3D,E and Fig. S3). From this analysis, the spring constants of single T4 GT7 DNA molecules are estimated as *k*_0_ = (20.1 ± 4.6) nN/m and* k*_HU_ = (75.4 ± 15.9) nN/m with 0 mM and 15 mM HU, respectively.

**Figure 4:**
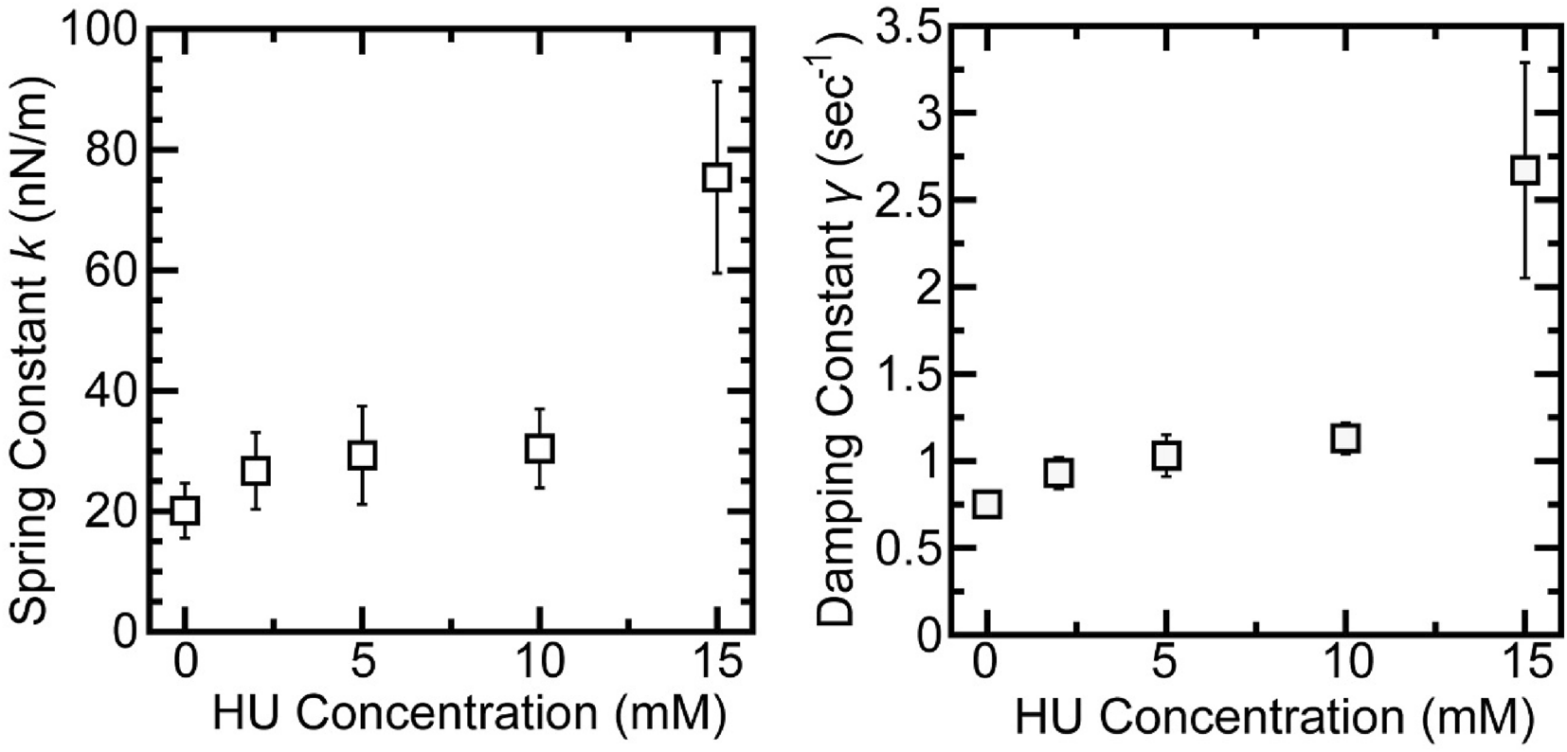
Viscoelasticity of single T4 GT7 DNA molecules in the presence of increasing concentrations of HU. The spring (left) and damping (right) constants were determined from the autocorrelation function of the intrachain thermal fluctuation observed by FM. Each experiment was repeated independently at least three times. Data are presented as the mean ± SD.

Figure 4 shows changes in spring *k* and damping γ constants at different HU concentrations, evaluated as described above. These data are also summarized in Table S1. In the presence of HU up to 10 mM, both *k* and γ were less sensitive to the HU concentration, but their values were 1.3 to 1.5 times higher than those in the absence of HU. We note that large increases of *k* and γ were caused at 15 mM HU, which corresponds to the HU concentration to cause the kinking structures in a prominent manner for the higher-order structure of DNA as revealed by AFM (see Fig. 2E).

### Minimum effect of HU on the secondary structure of DNA

**Figure 5:**
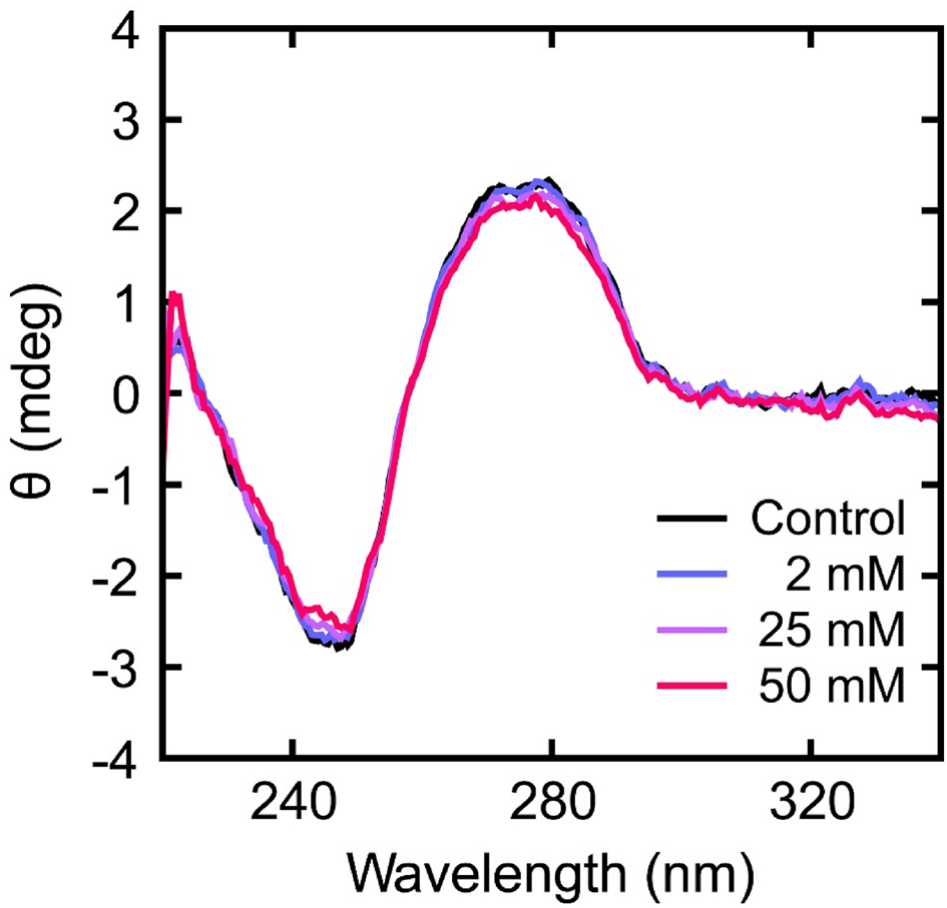
CD spectra of calf thymus (CT) DNA at different concentrations of HU. The concentration of CT DNA used was 30 μM for each sample.

We investigated the effects of HU on the secondary structure of DNA to clarify the mechanism of how HU generates partially-thick and kinked-branching structures on DNA and also why the viscoelasticity of single DNA changes in the presence of HU. Figure 5 shows the CD spectra of calf thymus (CT) DNA at different concentrations of HU. No apparent changes in the profile of the CD spectra were observed with increasing concentrations of HU up to 50 mM compared to untreated control. This means that the secondary structure of DNA was retained in the B-form, even in the presence of HU^26–30^. Thus, it becomes clear that HU causes an almost negligible effect on the secondary structure of DNA, in contrast to the marked effects on the higher-order structure of DNA (Figs. 2, 3, 4) and efficiency of gene expression (Fig. 1).

## Discussion

In this study, we found that HU exhibited a bimodal effect on the promotion and inhibition of gene expression, depending on its concentration (Fig. 1). AFM observations revealed that partially-thick and kinked-branching structures were generated by the addition of HU (Fig. 2, Fig. S1). Additionally, both the spring and damping constants increased when HU was added (Figs. 3, 4, Table S1) as measured by FM observation on the conformational Brownian motion. These constants provide useful insight concerning the mechanical properties of DNA. In contrast, HU exhibited an almost negligible effect on the secondary structure of DNA, as observed by CD measurements (Fig. 5). Our experimental results on the genetic activity, higher-order structure, and increasing spring/damping constants of DNA demonstrate that HU directly interacts with DNA. Previous research reports that the mechanism of action for the anticancer drug HU is to inhibit the enzyme RNR, which in turn blocks cellular DNA replication^11–16^. The results of the present study indicate that HU has additional effects on DNA, i.e., direct interaction with DNA. We expect this new insight will greatly contribute to a deeper understanding of the biological mechanism of HU. In the present study, we have obtained the evidence on the direct effect of HU on DNA, through the measurement of the mechanical property of large DNA by adapting T4 GT7 DNA (166 kbp; contour length 57 μm). It is well known that the persistence length of double-stranded DNA as the mechanical parameter of bending rigidity is 150 bp (~ 50 nm) in 0.1 M aqueous NaCl^31^. In other words, double-stranded DNA smaller than the persistence length behaves as a rigid rod, whereas larger DNA, more than 10^2^ times the persistence length, exhibits properties as an elongated random-coil chain. It has been reported that the persistence length of DNA tends to change sensitively in different solution conditions; for example, it drops to 30–20 nm by adding a multivalent cation. Additionally, it is also expected that twisting or writhing rigidity changes together with the change of bending rigidity, by inducing the change in asymmetric elasticity of large DNA^32^. Thus, it is regarded that the direct interaction of HU with double-stranded B-form DNA causes an apparent effect on its mechanical property through the integrated influence of the whole long DNA chain. With respect to the interaction energy of HU with water molecules in an aqueous solution, it was reported^33^ that the hydration enthalpy of HU is ca. 1.5 times greater than that of urea. It is well known that urea exhibits the potential to cause denaturation or change in the higher-order structure, of macromolecules in an aqueous environment. Thus, we may expect that the effect of HU to modify the hydration, as well as the manner of hydrogen bonding, is larger than that of urea. In other words, HU may modify the physicochemical properties of double-stranded DNA, such as the hydrogen bonding of deoxyribose moieties, the hydration of negatively charged phosphate moieties, and the hydrogen bonding between base pairs. These effects on the individual base-pair unit may accumulate for the long DNA segments above the size of persistence length and will cause an apparent change in the viscoelastic property of long DNA.

At present, the underlying mechanism of how the higher-order structure of DNA caused by HU concerns the bimodal effect on gene expression is still unclear. Interestingly, previous experiments^34–39^ using cell-free gene expression systems found polyamines exhibited bimodal effects on gene expression activity (i.e., promotion and inhibition at low (~ 100 µM) and high (above several hundred mM) concentrations, respectively). Polyamines induce a conformational transition of DNA from an elongated coil into a compact globule (coil-globule transition), accompanied by a large effective volume change on the order of 10^4^–10^5^, when the size of DNA is above several tens of kbp^34,35,38,40–42^. Thus, compact DNA prohibits the access of RNA polymerase and its substrate by inhibiting transcriptional activity. On the other hand, DNA molecules exhibit shrunken conformation, or a swelled state compared to the compact globule, at low polyamine concentrations, which corresponds to the polyamine concentrations to cause the promotion of gene expression^34,35,38^. Bimodal effects on gene expression have also been observed in a study measuring the effect of alcohols on in vitro gene expression. The efficiency of gene expression with a cell-free gene expression system increased around four to five times in the presence of a small amount of ethanol (2–3%)^23^. When the ethanol concentration was increased to ~ 10%, gene expression was completely inhibited. 2-Propanol exhibited a similar effect on gene expression but 1-propanol only inhibited gene transcription and marginally increased gene translation at low concentrations^23^. The spring constant and damping constant of a single DNA molecule increased slightly around 2% for the alcohols (ethanol, 1-propanol, and 2-propanol), and higher alcohol concentrations further increased these constants. Interestingly, similar effects between ethanol and HU were noticed for the enhancement of gene expression together with the changes in the mechanical properties of DNA. A slight increase in the spring and damping constants may provide a preferential working environment for RNA polymerase. Further studies to unveil the relationship between the activity of gene expression and higher-order conformation are awaited. It is also noted that genomic DNA molecules take poly-nucleosome structure through the binding with positively charged histones in eukaryotes. It would be important to investigate further the problem of how the direct effect of HU causes the change in the higher-order structure of genomic DNA and how the DNA activity is modified through its conformational changes.

Lastly, we would like to stress the usefulness of the experimental methodologies adopted in the present study to unveil the effect of chemical agents on the higher-order structural properties and gene expression of DNA. They can be used to (i) quantitatively evaluate the viscoelastic property of single DNA molecules in an aqueous solution without any external stress and (ii) measure the genetic activity of DNA by using cell-free gene expression. These methodologies would be useful for future studies that explore medicinal candidates for antitumor and other various diseases.

## Methods

### Materials

Hydroxyurea (HU; hydroxycarbamide), 2-mercaptoethanol (2-ME), and calf thymus DNA (CT DNA: 8–15 kbp) were purchased from Wako Pure Chemical Industries (Osaka, Japan). Tris-hydrochloride acid buffer (Tris–HCl; pH 7.4), 1 M MgCl_2,_ and T4 GT7 DNA (166 kbp, contour length 57 μm) were purchased from Nippon Gene (Tokyo, Japan). Plasmid DNA (Luciferase T7 Control DNA, 4331 bp) containing a firefly luciferase gene was purchased from Promega (Madison, WI, USA). The fluorescent cyanine dye, YOYO-1 (quinolinium, 1,1’- [1,3-propanediylbis[(dimethyliminio)-3,1-propanediyl]]bis[4-[(3- methyl-2(3H)-benzoxazolylidene)methyl]]-tetraiodide), was purchased from Molecular Probes, Inc. (Oregon, USA).

### Luciferase assay for gene expression

The cell-free luciferase assay was carried out using TnT (Rabbit Reticulocyte Lysate) T7 Quick Coupled Transcription/Translation System (Promega) according to the manufacturer’s instructions and previous reports^22,23,34–39^. Plasmid DNA (4331 bp) encoding a firefly luciferase gene with a T7 promoter sequence was utilized as the DNA template. The DNA concentration was 0.6 μM in nucleotide units. The reaction mixture including the DNA was incubated for 90 min at 30 °C with various concentrations of HU using a Dry Thermo Unit (TAITEC, Saitama, Japan). Luciferase expression was evaluated following the addition of luciferase assay substrate and buffer (Luciferase Assay Reagent, Promega) by detecting the light intensity around 565 nm with a luminometer (MICROTEC Co., Chiba, Japan).

### AFM observation

Atomic force microscopy (AFM) images were obtained using the scanning probe microscope, SPM-9700 (Shimadzu, Kyoto, Japan). T4 GT7 DNA (0.3 μM) was dissolved in buffer (10 mM Tris–HCl, 2 mM MgCl_2_, pH 7.4) and incubated for 12 min at room temperature (24 °C), then transferred onto a freshly cleaved mica surface. MgCl_2_ was used to attain the efficient absorption of DNA molecules onto the mica surface. It has been confirmed that the higher-order conformation exhibits negligible effect at such low (2 mM) concentration of MgCl_2_^18–20^.

Afterwards, the sample was rinsed with ultra-pure water, and dried with nitrogen gas. We imaged the mica surface and performed all measurements in the air using the tapping mode with AFM. The cantilever (OMCL-AC200TS-C3, Olympus, Tokyo, Japan) was 200 μm long with a spring constant of 9–20 N/m. The scanning rate was 0.4 Hz and images were captured using the height mode in a 512 × 512-pixel format. Obtained images were plane-fitted and flattened using the computer program supplied with the imaging module.

### FM observation

T4 GT7 DNA was dissolved in 10 mM Tris–HCl, pH 7.4, and 4% (v/v) 2-mercaptoethanol (2-ME; antioxidant) in the presence of various concentrations of HU (0–15 mM). To visualize individual DNA molecules by fluorescence microscopy (FM), 0.05 μM YOYO-1 (excitation/emission 491/509 nm) was added to the DNA solution. 2-ME was used to prevent the double-strand breaks during the fluorescence microscopic observation. Single DNA molecule observations were performed with the Axio Observer A1 inverted fluorescence microscope (Zeiss, Oberkochen, Germany), equipped with a 100 × objective lens. Images were obtained with a digital CMOS camera (Hamamatsu, Photonics, Hamamatsu, Japan). Images from the recorded videos at 30 frames per second were analyzed with ImageJ (Version 1.52, released 23 April 2018; National Institute of Mental Health, MD, USA). All observations were conducted at room temperature (24 °C). Based on the observation of consecutive time images, the long-axis length *L* of each DNA in solution was evaluated.

### CD measurements

Circular dichroism (CD) spectra of CT DNA were measured in the presence of HU in 10 mM Tris–HCl, pH 7.4, on a J-820 spectropolarimeter (JASCO, Tokyo, Japan). The DNA concentration was 30 μM in nucleotide units for all the CD spectra measurements. The cell path length was 1 cm. Data were obtained every 0.5 nm between 220 and 340 nm at a scan rate of 50 nm/min and were accumulated 3 times.

### Supplementary Information

Supplementary Information.

## Data Availability

All data presented in this study are contained within the article and Supplementary Materials.
