# Supplementary material for: Characteristic effect of hydroxyurea on the higher-order structure of DNA and gene expression
Source: Sci Rep. 2024 Jun 15;14:13826. doi: 10.1038/s41598-024-64538-y (PMC11180115; doi:10.1038/s41598-024-64538-y)
Supplement: Supplementary file 1 — Supplementary Information. [file 41598_2024_64538_MOESM1_ESM.pdf]

Supplementary Information

Characteristic Effect of Hydroxyurea on the Higher-Order Structure of DNA and Gene Expression

Haruto Ogawa[1], Takashi Nishio[1,2], Yuko Yoshikawa[1], Koichiro Sadakane[1], Takahiro Kenmotsu[1], Tomoyuki Koga[3], Kenichi Yoshikawa[1,4,*]

[1]Faculty of Life and Medical Sciences, Doshisha University, Kyoto, 610-0394, Japan
[2]Cluster of Excellence Physics of Life, TUD Dresden University of Technology, Dresden, 01307, Germany
[3]Department of Molecular Chemistry and Biochemistry, Faculty of Science and Engineering, Doshisha University, Kyoto, 610-0321, Japan
[4]Center for Integrative Medicine and Physics, Institute for Advanced Study, Kyoto University, Kyoto, 606-8501, Japan

* Email: keyoshik@mail.doshisha.ac.jp

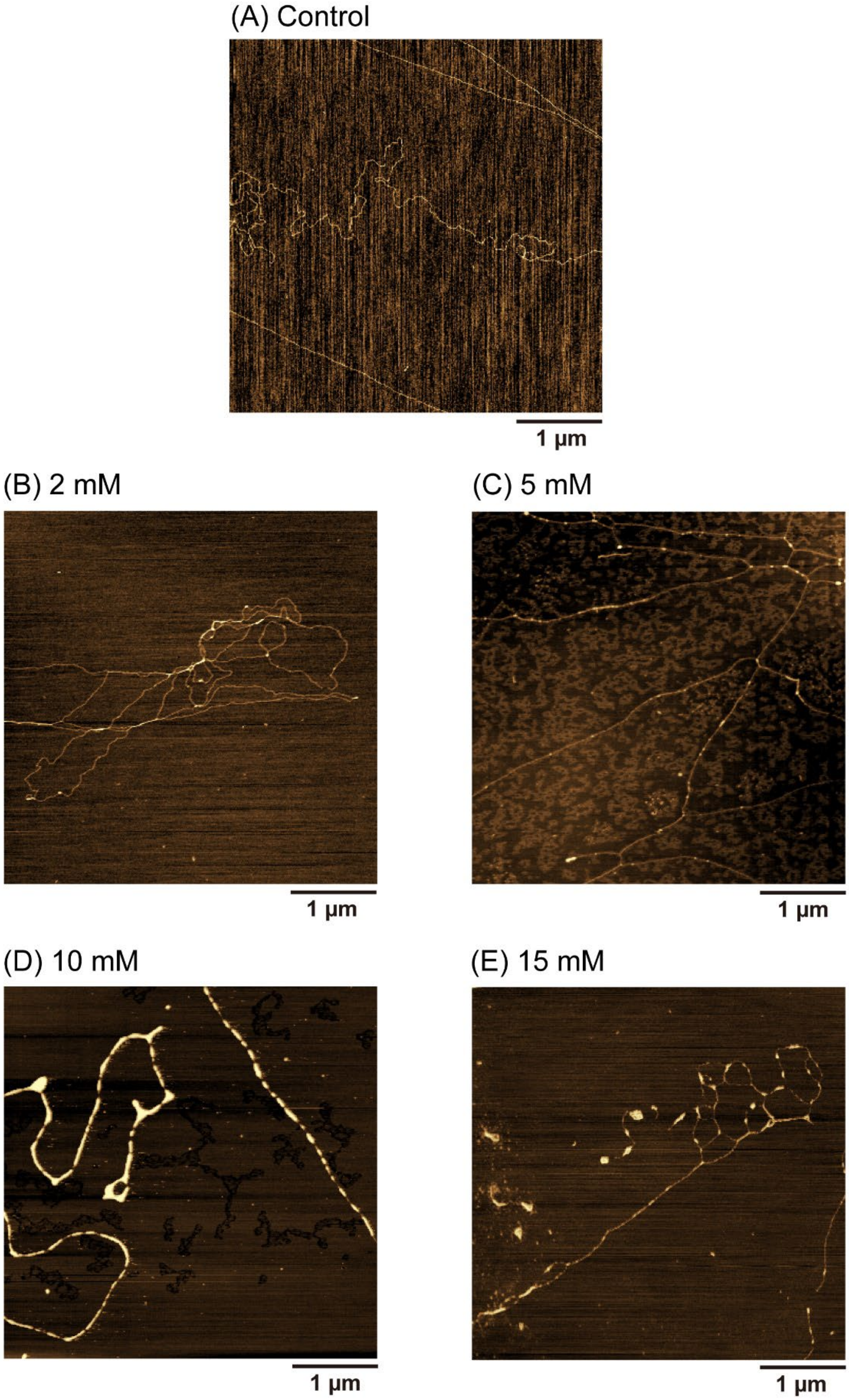

Figure S1. Additional AFM images of T4 GT7 DNA ((A) 0 mM HU (Control), (B) 2 mM HU, (C) 5 mM HU, (D) 10 mM HU, (E) 15 mM HU) in 2 mM $MgCl_2$.

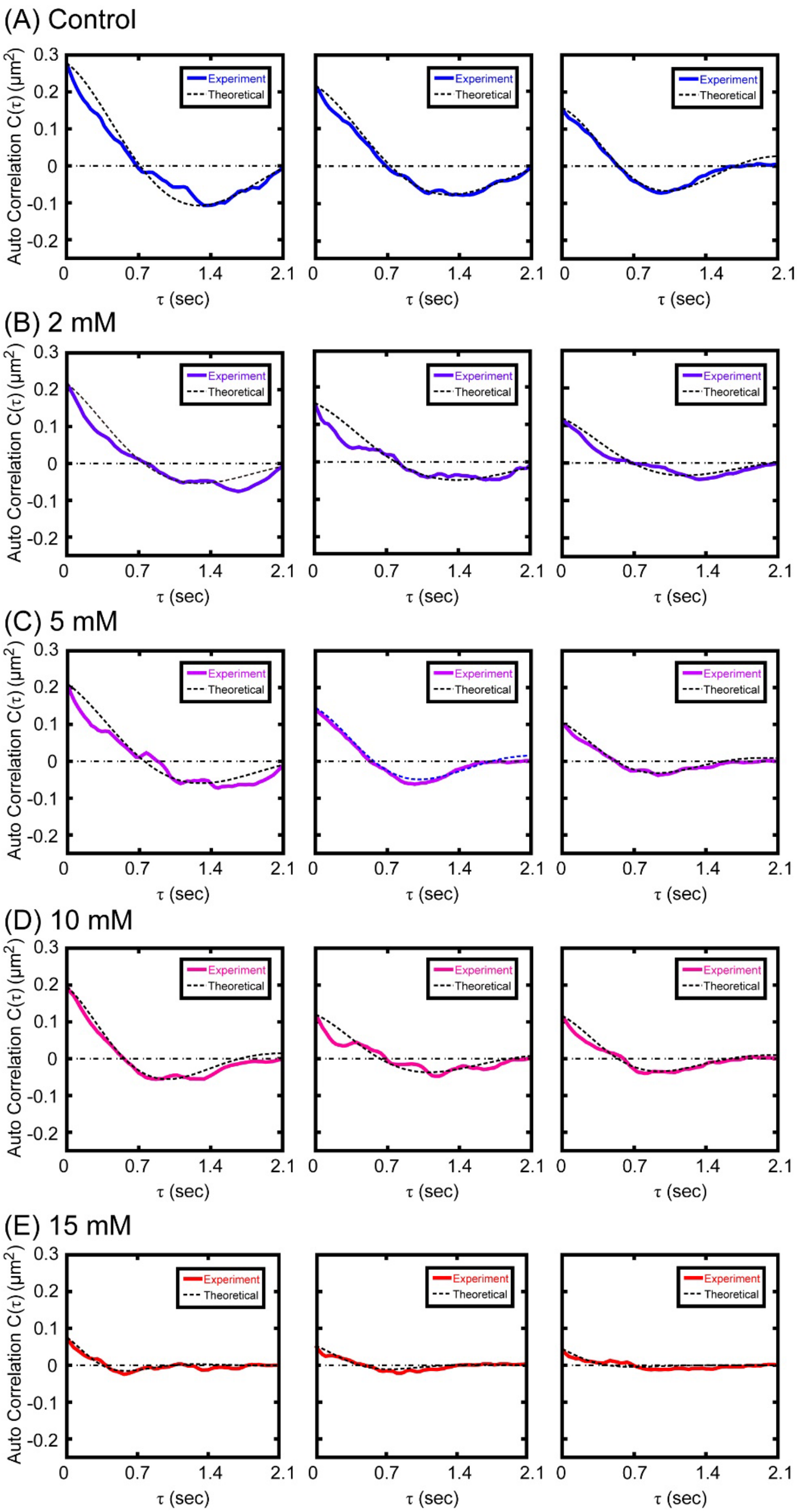

Figure S2. Autocorrelation of the time-dependent fluctuation of the long-axis length of single T4 GT7 DNA molecules in the presence of 0 (Control), 2, 5, 10, and 15 mM HU.

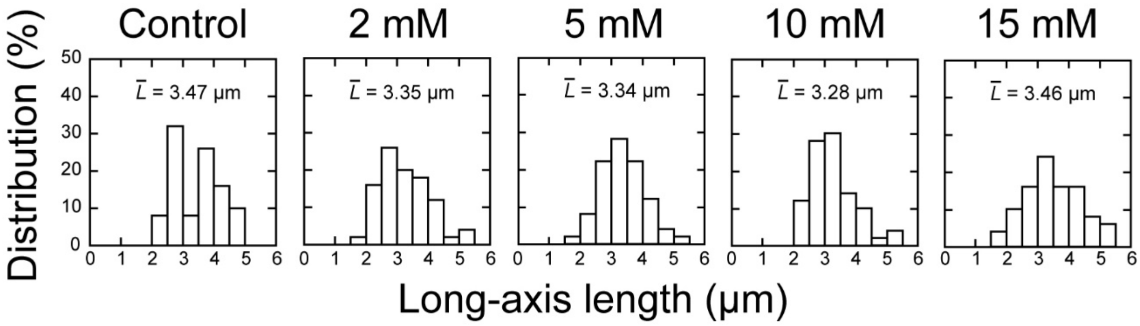

Figure S3. Distribution of the long-axis length L of T4 GT7 DNA observed by FM. At each condition, 50 DNA molecules were measured and the mean values, $\bar{L}$, are given in the boxes.

Table S1. Viscoelastic parameters evaluated from the analysis of the interchain fluctuation of single T4 GT7 DNA molecules.

		C(0) (μm^2)	Spring constant k (nN/m)	Damping constant γ (sec^{-1})
	Control	0.22±0.05	20.1±4.6	0.75±0.04
HU	2 mM	0.17±0.04	26.7±6.4	0.93±0.09
	5 mM	0.15±0.05	29.3±8.2	1.03±0.12
	10 mM	0.15±0.04	30.4±6.6	1.13±0.09
	15 mM	0.06±0.01	75.4±15.9	2.67±0.62
